# Use of a Biodegradable, Contrast-Filled Rectal Spacer Balloon in Intensity-Modulated Radiotherapy for Intermediate-Risk Prostate Cancer Patients: Dosimetric Gains in the BioPro-RCMI-1505 Study

**DOI:** 10.3389/fonc.2021.701998

**Published:** 2021-08-26

**Authors:** Igor Latorzeff, Eric Bruguière, Emilie Bogart, Marie-Cécile Le Deley, Eric Lartigau, Delphine Marre, David Pasquier

**Affiliations:** ^1^Department of Radiotherapy, Clinique Pasteur, Toulouse, France; ^2^Department of Imaging, Clinique Pasteur, Toulouse, France; ^3^Methodology and Biostatistics Unit, Centre Oscar Lambret, Lille, France; ^4^Academic Department of Radiation Oncology, Centre Oscar Lambret, Lille, France; ^5^CRIStAL UMR CNRS 9189, Lille University, Lille, France; ^6^Department of Physics, Clinique Pasteur, Toulouse, France

**Keywords:** prostate cancer, intermediate risk group, intensity-modulated radiotherapy, prospective study, spacer with biodegradable contrast-filled rectal balloon, organs at risk, dosimetric analyses, quality of life

## Abstract

**Background/purpose:**

Dose-escalated external beam radiotherapy (RT) is effective in the control of prostate cancer but is associated with a greater incidence of rectal adverse events. We assessed the dosimetric gain and safety profile associated with implantation of a new biodegradable rectal spacer balloon.

**Materials/methods:**

Patients scheduled for image-guided, intensity-modulated RT for intermediate-risk prostate cancer were prospectively included in the French multicenter BioPro-RCMI-1505 study (NCT02478112). We evaluated the dosimetric gain, implantation feasibility, adverse events (AEs), and prostate-cancer-specific quality of life associated with use of the balloon spacer.

**Results:**

After a scheduled review of the initial recruitment target of 50 patients by the study’s independent data monitoring committee (IDMC), a total of 24 patients (including 22 with dosimetry data) were included by a single center between November 2016 and May 2018. The interventional radiologist who implanted the balloons considered that 86% of the procedures were easy. 20 of the 24 patients (83.3%) received IMRT and 4 (16.7%) received volumetric modulated arc therapy (78-80 Gy delivered in 39 fractions). The dosimetric gains associated with spacer implantation were highly significant (p<0.001) for most variables. For the rectum, the median (range) relative gain ranged from 15.4% (-9.2−47.5) for D20cc to 91.4% (36.8−100.0) for V70 Gy (%). 15 patients (62%) experienced an acute grade 1 AE, 8 (33%) experienced a late grade 1 AE, 1 (4.2%) experienced an acute grade 2 AE, and 3 experienced a late grade 2 AE. No grade 3 AEs were reported. Quality of life was good at baseline (except for sexual activity) and did not markedly worsen during RT and up to 24 months afterwards.

**Conclusion:**

The use of a biodegradable rectal spacer balloon is safe, effective and associated with dosimetric gains in modern RT for intermediate-risk prostate cancer.

## Introduction

A number of randomized clinical trials have notably demonstrated that dose-escalated external beam radiotherapy (RT) can effectively achieve good biochemical and clinical outcomes in prostate cancer (1–6). In the multicenter Medical Research Council RT01 trial, patients were randomized to conformal RT with either 64 or 74 Gy (2 Gy/session) plus 3 to 6 months of neoadjuvant hormone therapy; the 5-year biochemical relapse–free survival rate was 71% in the 74 Gy group and 60% in the 64 Gy group (p=0.0007) (3). Likewise, the GETUG 06 trial showed that dose escalation from 70 to 80 Gy provided a better 5-year biochemical outcome but slightly more adverse events (AEs) (1). However, the anatomic proximity between the prostate, the urinary tract and the rectum means that the latter are also exposed to the toxic effects of ionizing radiation. Hence, dose escalation is associated with a higher relapse-free survival rate but also with a greater frequency of urinary tract and rectal AEs and erectile dysfunction. The development of modern, intensity-modulated RT (IMRT) enabled escalation of the prostate dose to 78 Gy with the same risk of rectal toxicity as three-dimensional conformal RT at 70 Gy (3). Furthermore, the use of volume-modulated arc therapy has shortened treatment times without sacrificing tissue coverage (7, 8).Lastly, irradiation of the urinary tract and rectum can be minimized by targeting the dose to the prostate as accurately as possible with using image-guided RT (IGRT). In a comparative study, the use of IGRT was associated with a lower rate of grade ≥2 urinary tract AEs at 3 years (10.4%, *vs.* 20% in a control group) (5).

Despite these technical advances, however, the dose delivered to the rectum (*via* external beam RT or brachytherapy) remains a limiting factor in dose escalation. A number of researchers reasoned that the incidence and severity of rectal AEs could be reduced by increasing the distance between the prostate and the rectum *via* the insertion or injection of spacers made of biodegradable material [e.g. hyaluronic acid (HA)] or non-biodegradable material [e.g. polyethylene glycol (PEG)] into the perirectal fat. Indeed, the use of spacers is associated with less rectal AEs (9–11). By way of an example, 222 patients with stage T1 or T2 prostate cancer and undergoing image-guided IMRT (79.2 Gy in 1.8-Gy fractions) were randomized to spacer implantation or no implantation (12, 13). The incidence of rectal AEs 3 to 15 months after treatment was significantly lower in the spacer group (2.0%) than in the control group (7.0%; p=0.04). Furthermore, bowel-related quality of life (QoL) 6, 12, and 15 months after the end of IMRT was significantly better in the spacer group (12, 13).

The ProSpace^®^ biodegradable fillable balloon (BioProtect Ltd, Tzur Yigal, Israel) is a rectal spacer with confirmed safety and efficacy in preclinical and clinical studies (14–19).

Although the insertion procedure is slightly more invasive than for HA and PEG spacers (a small perineal incision and a special dilator and sheath are required), inflation of the balloon with sterile diluted iodine contrast solution (or physiological saline solution, if iodine is contraindicated) avoids the potential lateral and craniocaudal dispersion of spacer material (18). In a Phase II multicenter study, the mean ± standard deviation (SD) prostate-rectum distance was 0.22 ± 0.2 cm before insertion and 2.47 ± 0.47 cm after insertion; this distance was maintained during RT (20).

The present prospective, interventional, multicenter study was designed to assess the dosimetric gain, implantation procedure, and acute and late AEs associated with use of the contrast-filled ProSpace^®^ balloon for better image-guided targeting in patients undergoing IMRT of intermediate-risk prostate cancer (16). Here, we report the final results for the primary efficacy criterion (dosimetric gain) and some of the secondary criteria, together with intermediate results for other secondary criteria (notably QoL and safety).

## Methods and Materials

The study’s rationale and protocol (including the study objectives, inclusion and exclusion criteria, device characteristics, device implantation, dosimetric criteria, safety evaluation and patient-reported outcomes) have been described in detail elsewhere (16). Briefly, adult patients scheduled for IGRT (with cone-beam CT) and IMRT (78 G, 2 Gy/fraction) for intermediate-risk prostate cancer [according to the D’Amico classification (21)] were prospectively screened for eligibility in six French cancer centers. The study’s main inclusion and exclusion criteria are listed in **Supplementary Table 1**, and the study visits and procedures are summarized in **Supplementary Table 2**. The primary objective was to evaluate the dosimetric gain for the organs at risk (OAR) associated with use of the ProSpace^®^ biodegradable balloon. The secondary objectives were to evaluate (i) the technical feasibility of the balloon’s implantation, (ii) AEs (evaluated according to the National Cancer Institute – Common Terminology Criteria for Adverse Events (CTCAE, version 4.0; https://ctep.cancer.gov/protocoldevelopment/electronic_applications/ctc.htm#ctc_40), (iii) the time interval between implantation and the initiation of radiotherapy and the relationship with implantation-related complications, (iv) the association between ProSpace^®^ use and treatments for acute proctitis and (v) QoL (using the European Organisation for Research and Treatment of Cancer (EORTC) score QoL self-questionnaire (QLQ-C30) and the prostate-cancer-specific PR25 module (22, 23). “Early” AEs were defined as those arising within 6 months (rather than 3 months, in the CTCAE) of RT.

The dosimetry plans before and after ProSpace^®^ implantation were calculated using Eclipse treatment planning software (Varian, Palo Alto, CA). For the purposes of the present publication, data were collected and doses were reported and analyzed using the Aquilab SharePlace platform (including ArtiviewTM 3.20.1 software) from Aquilab SAS (Loos Les Lille, France). Aquilab SAS also managed the study’s electronic case report form, the study database, and the on-line patient self-questionnaires.

In all cases, the ProSpace^®^ was implanted in an operating room by the same interventional radiologist. During inflation of the balloon with saline solution, the investigators added 1 ml of iodine contrast enhancer in order to improve the IGRT procedure and enhance the balloon’s delineation on the planning CT. The implantation of a contrast-filled ProSpace^®^ balloon has been described in detail by Vanneste et al. (18).

The study was approved by an institutional review board (*Comité de Protection des Personnes Nord Ouest I*, Lille, France; reference: 13/10/2016) and registered at ClinicalTrials.gov (NCT02478112). All included patients received information on the study’s objectives and procedures and gave their written consent to participation.

## Results

### Study Population and Treatment

A total of 24 patients were included in the study between November 28^th^, 2016, and May 28^th^, 2018. Initially, 50 patients were planned for accrual but an intermediate, scheduled review by the study’s independent data monitoring committee (IDMC) stopped patient enrolment after the first 24, since the primary objective had been achieved. Hence, although the study had a multicenter design, all 24 patients came from a single cancer center (Toulouse, France). The characteristics of the study population on inclusion are summarized in **Table 1**. All patients were evaluated with MRI before study entry and the cancer was staged as T2 in all cases. Two patients lacked dosimetry data after ProSpace^®^ implantation. Hence, 22 patients were included in the dosimetry analysis.

**Table 1:** Characteristics of the study population on inclusion.

Variables (n=24)			Characteristics (n=24)		
**Age (years)**			**Medical history**		
median (range)	75.5	(61.0−81.0)	infectious disease	0	0%
mean ± SD	74.1	± 5.2	digestive tract disease	1*	4.2%
**Clinical T stage**			prostate resection	5	20.8%
T1c	4	16.7%	cardiovascular disease	15	62.5%
T2a	16	66.7%	type II diabetes	3	12.5%
T2b	2	8.3%	pelvic surgery	0	0%
T2c	2	8.3%	**Androgen deprivation therapy**	11	45.8%
**N0 status**	24	100.0%	Medications other than androgen deprivation therapy^*^	16	66.7%
**M0 status**	24	100.0%	**Anticoagulants**	0	0%
**Initial serum PSA (ng/ml)**			**Biopsies**		
median (range)	7.1	(0.6−19.6)	**Number of biopsy cores**		
mean ± SD	8.0	± 4.2	median (range)	14.5	(5.0−24.0)
**Prostate volume (cc)**			mean ± SD	14.8	± 4.6
median (range)	34.0	(15.0−89.0)	**Number of positive biopsy cores**		
mean ± SD	36.7	± 17.2	median (range)	4.0	(1.0−13.0)
**ECOG PS = 0**	24	100.0%	mean ± SD	4.8	± 3.2
**Total Gleason score**			**Total length of positive biopsies (mm)**		
6	2	8.3%	median (range)	20.0	(1.0−50.0)
7	22	91.7%	mean ± SD	20.4	± 12.8
			**Proportion of positive biopsies (%)**		
			median (range)	30.0	(7.1−86.7)
			mean ± SD	35.3	± 22.1
			**Side(s) invaded**		
			left only	7	29.2%
			right only	4	16.7%
			left and right	13	54.2%

SD, standard deviation; PSA, prostate-specific antigen; ECOG PS, Eastern Cooperative Oncology Group Performance Status; BMI, body mass index.

*medications other than androgen deprivation therapy included treatments for diabetes and other metabolic diseases, arterial hypertension and other cardiovascular diseases, gout, allergy, asthma, arthritis, insomnia, stress, and glaucoma.

**Figure 1:**
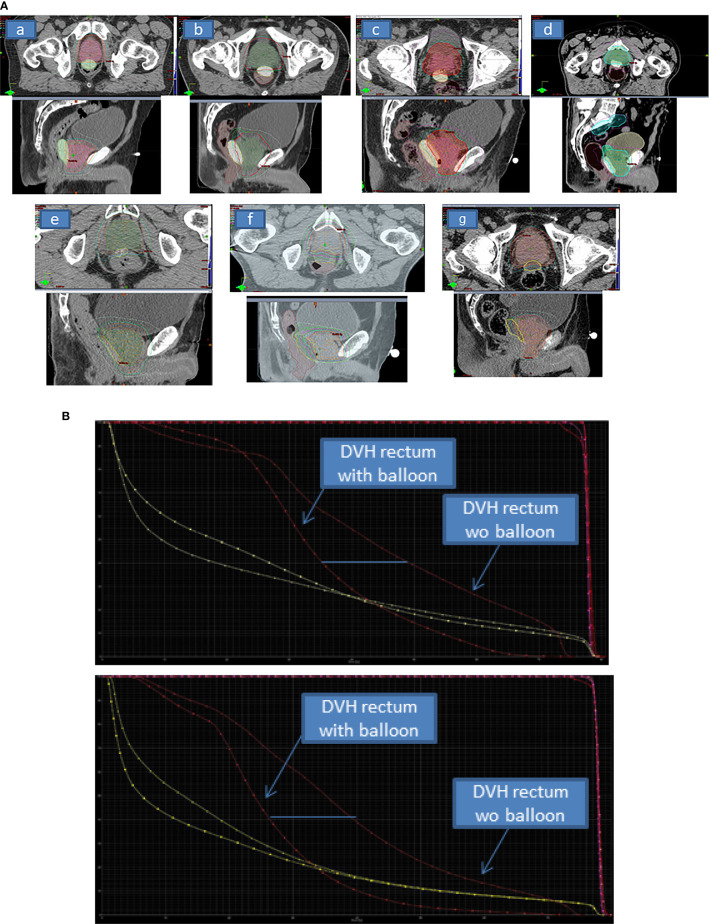
**(A)** The planning CT axial and sagittal views of delineated volumes of interest (prostate gland and the ProSpace^®^.biodegradable balloon) showing good quality of delineation with iodinated contrast-filling balloon (a–g). For 1 patient (e) iodine contrast product was too much diluted. For 2 patients (f, g), the procedure was performed without iodine contrast enhancement, and so delineation of the balloon was less easy. **(B)** The dose-volume histogram (DVH) for the rectum pre-balloon and post-balloon for 2 patients, showing the dosimetric benefit achieved with the balloon. The DVH for bladder (also shown, in yellow) is not modified. With regard to the clinical target volume and the planned treatment volume (shown in pink and red, respectively), balloon implantation was associated with greater homogeneity.

21 of the 24 patients received a contrast-filling balloon, and 3 patients received a balloon without contrast (iodine allergy: n=2; protocol deviation: n=1). The interventional radiologist considered that the implantation was easy or very easy in 19 of the 22 cases (86%). Difficulties were noted in three cases (14%): incomplete inflation of the balloon due to resistance; difficulty crossing the perineal region and slight displacement of the balloon at the end of the inflation; failure to inflate the balloon (though a second balloon inflated with no problems) (**Figure 1A**). Further results for the implantation procedures are given in **Table 2**.

**Table 2:** Characteristics of the ProSpace^®^ balloon implantation.

Variables (n=24)		
**Time interval between implantation and the start of RT (days)**		
median (range)	23.0	(21.0−35.0)
mean ± SD	24.5	± 4.2
*missing data*	*6*	
**Type of anesthesia**		
general	22	91.7%
local	2	8.3%
**Duration of the surgical session (min)**		
median (range)	36.0	(13.0−64.0)
mean ± SD	33.5	± 12.9
**Duration of the implantation (min)**		
median (range)	14.0	(1.0−23.0)
mean ± SD	14.2	± 6.2
* missing data*	*1*	

RT, radiotherapy; SD, standard deviation.

With regard to treatment, 20 of the 24 patients (83.3%) received IMRT and 4 (16.7%) received volumetric modulated arc therapy. The median (range) duration of RT was 58.5 days (55.0−68.0), and the mean ± SD duration was 59.3 ± 3.5 days. In all cases, contrast-free CT was used for contouring. The treatment volume included the seminal vesicles in 22 of the 24 cases (91.7%) and the pelvis in 2 (8.3%) to a dose of 46 Gy. In all 24 cases, the total planned dose was 78 Gy delivered in 39 fractions.

### Dosimetry Data

As mentioned above, dosimetry data before and after ProSpace^®^ implantation were available for 22 patients (**Table 3**). The median dosimetric gains (whether expressed in absolute or relative terms) associated with ProSpace^®^ implantation were highly significant (p<0.001) for the majority of the dosimetric variables. For the rectum, the median (range) relative gain ranged from 15.4% (-9.2−47.5) for D20cc to 91.4% (36.8−100.0) for V70 Gy (the percentage volume of the rectum receiving 70 Gy radiation). Non-significant differences were observed for Dmax (rectum), V50% (rectum), V70% (bladder, cc), V60% (bladder, cc) and V50% (bladder, cc) (**Figure 1B**). The absolute dosimetric gains were significant for D2.5cc, D5cc, D10cc, D15cc, D20cc, V70 Gy, V90%, V80%, and V60% (all p<0.001) (**Table 3**).

**Table 3:** Dosimetry parameters before and after ProSpace^®^ balloon implantation.

Variables (n=22)	Before Balloonimplantation		After Balloonimplantation		Relative Gain (%)		Absolute Gain		p*
**Dmax - rectum (Gy)**									0.067
median (range)	76.2	(75.1−77.1)	75.8	(66.8−77.4)	0.4	(-2.3−12.1)	0.3	(-1.7−9.2)	
mean ± SD	76.1	± 0.5	75.3	± 2.2	1.1	± 2.9	0.8	± 2.2	
**D2.5cc - rectum (Gy)**									**<0.001**
median (range)	73.6	(71.8−73.9)	63.5	(47.2−73.8)	13.7	(- 0.1−35.0)	10.1	(- 0.1−25.4)	
mean ± SD	73.4	± 0.5	61.3	± 8.1	16.5	± 10.7	12.1	± 7.8	
**D5cc - rectum (Gy)**									**<0.001**
median (range)	71.9	(66.0−73.3)	56.6	(40.7−70.8)	20.9	(2.8−40.1)	14.8	(2.0−27.8)	
mean ± SD	71.4	± 2.0	55.6	± 8.0	22.1	± 10.3	15.7	± 7.2	
**D10cc - rectum (Gy)**									**<0.001**
median (range)	65.0	(52.7−69.4)	50.7	(34.0−59.4)	20.9	(9.6−44.4)	13.1	(6.1−30.2)	
mean ± SD	63.7	± 4.5	49.1	± 7.6	22.8	± 10.8	14.6	± 7.1	
**D15cc - rectum (Gy)**									**<0.001**
median (range)	56.9	(43.9−63.1)	45.7	(30.0−54.0)	17.0	(2.6−46.5)	10.3	(1.4−29.3)	
mean ± SD	55.7	± 5.5	44.6	± 7.2	19.6	± 13.1	11.2	± 7.8	
**D20cc - rectum (Gy)**									**<0.001**
median (range)	50.9	(36.6−58.7)	41.1	(26.9−51.3)	15.4	(-9.2−47.5)	8.6	(-3.7−27.9)	
mean ± SD	49.3	± 6.3	40.9	± 6.9	16.0	± 16.2	8.4	± 8.4	
**V90% - rectum (cc)**									**<0.001**
median (range)	6.6	(3.5−9.3)	0.6	(0.0−5.3)	90.2	(32.5−100.0)	5.1	(2.5−8.4)	
mean ± SD	6.5	± 1.6	1.1	± 1.3	84.2	± 17.1	5.3	± 1.5	
**V80% - rectum (cc)**									**<0.001**
median (range)	11.4	(6.2−15.8)	2.7	(0.1−8.4)	78.0	(26.4−99.1)	7.5	(3.0−15.4)	
mean ± SD	11.3	± 2.6	2.9	± 2.3	74.4	± 19.5	8.3	± 2.9	
**V60% - rectum (cc)**									**0.001**
median (range)	24.1	(13.1−35.3)	14.0	(2.6−31.3)	37.1	(-21.6−88.1)	9.5	(-5.6−29.0)	
mean ± SD	23.3	± 6.0	13.8	± 8.4	39.9	± 34.0	9.5	± 8.8	
**V50% - rectum (cc)**									0.058
median (range)	30.9	(18.5−50.3)	22.2	(5.9−62.6)	16.6	(-67.7−81.2)	5.6	(-25.3−37.7)	
mean ± SD	31.9	± 9.1	25.3	± 14.2	18.4	± 39.3	6.6	± 14.6	
**V70 Gy - rectum (cc)**									**<0.001**
median (range)	6.7	(3.5−9.5)	0.6	(0.0-5.3)	90.0	(32.5−100.0)	5.2	(2.6−8.6)	
mean ± SD	6.6	± 1.7	1.2	± 1.3	84.1	± 17.1	5.4	± 1.5	
**V70 Gy - rectum (%)**									**<0.001**
median (range)	9.7	(5.2−19.6)	0.7	(0.0−8.1)	91.4	(36.8−100.0)	8.5	(4.7−15.6)	
mean ± SD	10.7	± 3.9	1.8	± 2.2	85.2	± 16.4	8.9	± 3.1	
**V70% - bladder (cc)**									0.10
median (range)	59.0	(18.5−103.7)	49.6	(21.5−95.3)	8.2	(-38.9−62.7)	4.9	(-22.5−39.8)	
mean ± SD	58.3	± 18.9	53.2	± 20.4	7.7	± 21.3	5.2	± 12.1	
**V60% - bladder (cc)**									0.22
median (range)	72.0	(23.6−126.5)	64.0	(27.5−111.0)	8.1	(-41.2−62.0)	6.2	(-27.5−58.3)	
mean ± SD	74.0	± 22.5	67.3	± 22.5	6.6	± 22.3	6.7	± 17.0	
**V50% - bladder (cc)**									0.39
median (range)	90.8	(31.3−147.1)	88.1	(36.4−133.3)	5.6	(-41.2−63.4)	5.7	(-31.1−92.3)	
mean ± SD	93.3	± 28.8	85.5	± 25.3	4.5	± 23.0	7.8	± 24.5	
**Homogeneity of the prostate CTV (10^3^)**									**0.002**
median (range)	29.5	(22.0−70.0)	21.5	(14.0−146.0)	29.4	(-108.6−56.3)	8.0	(-76.0−19.0)	
mean ± SD	31.2	± 9.9	26.8	± 26.9	20.4	± 33.8	4.4	± 18.8	

DXcc, dose delivered to X cc of the indicated anatomic structure; VX%, volume receiving X% of the prescribed dose; V70 Gy, volume of the indicated anatomic structure receiving 70 Gy; CTV, clinical target volume. *calculated for the relative gain, using Wilcoxon’s test. Bold values: p < 0.05.

With regard to safety, 5 of the 24 patients (21%) did not experience any AEs, 15 (62%) experienced a grade 1 AE, and 4 (17%) experienced a grade 2 AE. No grade 3 AEs were reported. Sixteen patients (67%) experienced an acute AE (grade 1 or 2), and 11 (46%) experienced a late AE. Urinary frequency was the most common acute AE (grade 1 for 13 patients and grade 2 for 1) and the most common late AE (grade 1 for 5 patients and grade 2 for 2). Only one AE (proctitis) was considered by an investigator to be related to ProSpace^®^ implantation, although the event started a week after the first RT session and a month after the implantation. As this was the only AE though to be related to ProSpace^®^ implantation, we were unable to assess the relationship between complications on one hand and the time interval between implantation and the start of RT on the other.

Before and after RT, the median International Prostate Symptom Score (IPSS) ranged from 3 to 5 (**Table 4**). The IPSS increased during RT, and 5 patients had experienced severe symptoms at this point.

**Table 4:** Prostate symptoms before, during and after RT, as rated on the IPSS.

IPSS	Baselinen=21		Start of RTn=20		Mid-RTn=22		End of RTn=23		3 monthspost-RTn=23		6 monthspost-RTn=23		12 monthspost-RTn=24		24 monthspost-RTn=12	
Median (range)	5.0	(1.0–17.0)	3.5	(0.0–18.0)	7.0	(2.0–28.0)	11.0	(2.0–28.0)	4.0	(0.0–15.0)	3.0	(0.0–18.0)	3.5	(0.0–15.0)	3.5	(1.0–13.0)
Mean ± SD	5.4	± 3.9	4.8	± 4.5	9.8	± 6.6	12.2	± 8.2	5.0	± 3.5	5.0	± 4.3	5.3	± 4.5	5.8	± 4.4
Mild symptoms (0-7), n (%)	16	76.2%	15	75.0%	12	54.5%	9	39.1%	18	78.3%	17	73.9%	17	70.8%	7	58.3%
Moderate symptoms (8-19), n (%)	5	23.8%	5	25.0%	8	36.4%	9	39.1%	5	21.7%	6	26.1%	7	29.2%	5	41.7%
Severe symptoms (20-35), n (%)	0	0.0%	0	0.0%	2	9.1%	5	21.7%	0	0.0%	0	0.0%	0	0.0%	0	0.0%

IPSS, International Prostate Symptom Score; RT, radiotherapy; SD, standard deviation.

### Quality of Life

**Figure 2:**
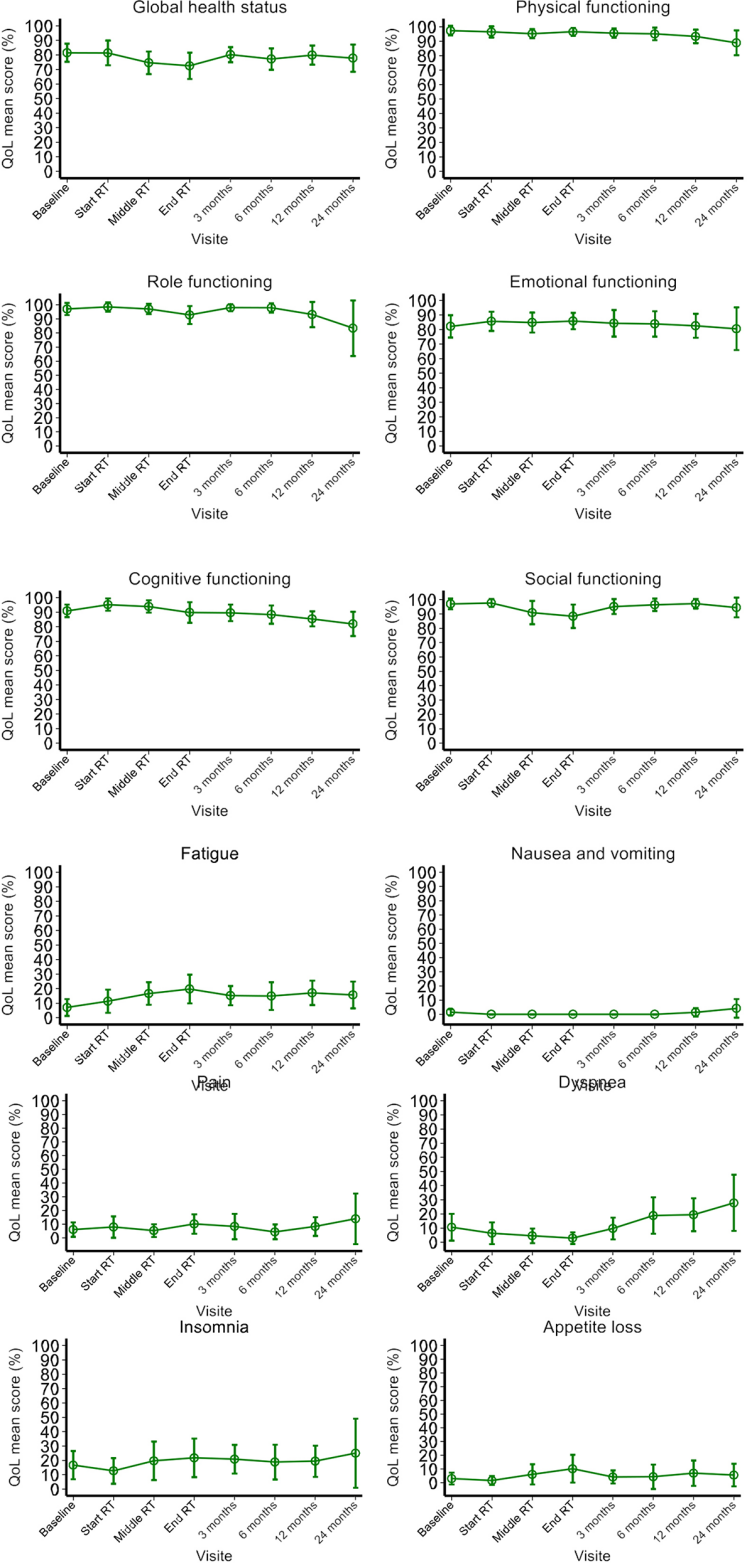
Prostate cancer-specific QoL before, during and after RT, as assessed with the EORTC QLQ-C30 and the EORTC QLQ-PR25 self-questionnaires.

At baseline, the QLQ-C30 and PR25 questionnaires gave mean values of >80 for the “functioning” domains and <20 for the symptom domains. The exception was the PR25 sexual activity score, with a mean (range) value of 66.7 (0 – 100) at baseline. We then observed (i) a slight worsening of the scores for fatigue, loss of appetite, constipation and diarrhea, and urinary symptoms and problems during and immediately after RT, and (ii) worsening of the score of dyspnea during the post-RT follow-up (**Figure 2**). The other domain scores remained stable during RT and up to 24 months thereafter.

## Discussion

In the prospective BioPro-RCMI-1505 study, we evaluated the routine use of a relatively new rectal spacer as part of a modern IMRT/IGRT protocol. Our present results indicate that the balloon is a safe, efficacious adjunct to IMRT for prostate cancer; it was associated with dosimetric gains that help to spare the wall of the rectum from the effects of a higher dose to the prostate. Placement of the balloon spacer was relatively easy for physicians with experience of transrectal prostate procedures. Filling the balloon with contrast solution facilitates delineation of the spacer volume on the planning CT (18). The level of patient satisfaction was high, and the patients reported good QoL before and after the procedure. The delivery of a high dose of radiation (~78 Gy) to the prostate in IMRT increases the likelihood of tumor control; the percentage of patients with a grade 2 gastro-intestinal AE ranges from 1% to 23%, and the percentage with a grade 3 AE is very low (0% to 3%) (2). In a retrospective study performed in the USA, the combination of image guidance with IMRT dose escalation was associated with a low proportion of patients with late grade 2 genitourinary tract AEs (5). In a randomized phase III study of a PEG hydrogel spacer (SpaceOAR, Augmenix, Inc., Bedford, MA) in modern IMRT/IGRT for prostate cancer, the dosimetric gains were associated with a lower incidence of late grade ≥1 rectal AEs (24).

When considering the primary objective, we found that use of the balloon spacer resulted in statistically significant dosimetric gains for the rectum. Moreover, the adjunction of a spacer between the prostate and the rectum increased CTV homogeneity in our cohort. This result could lead to a difference for bladder dose coverage with IMRT dosimetry. Basically to spare the rectum wall without spacer, IMRT planning is performed with CTV heterogeneity with the maximum dose to the prostate located at the anterior part of the prostate, close to the bladder neck. Adding spacer allows better dose CTV homogeneity and we reported bladder dose distribution V70%, V60% and V50% differences but these findings didn’t reach statistical significance level.

Our present dosimetric and safety results for a balloon spacer are in line with the literature data for PEG and HA gel spacers (12, 24, 25). In the randomized study of a PEG gel spacer described by Karsh et al., the median rectal V70 dose was 2.3% in the spacer and 10.5% in the control group; this corresponded to a relative reduction of 78% (p ≤ 0.0001). There were no intergroup differences in the incidence of acute grade ≥2 rectal AEs (4.1% *vs.* 4.2% in the spacer and control groups, respectively; p=0.5) or acute grade ≥2 urinary tract AEs (37.8% *vs* 44.4%, p=0.5). The incidence of late grade ≥1 rectal AEs at 37 months was significantly lower in the spacer arm (2%) than in the control arm (9%; p<0.03). Moreover, none of the patients in the spacer group experienced a late grade ≥2 rectal AE (24). QoL was significantly better in the spacer group; at 3 years, the proportions of men in the control and spacer groups experiencing a QoL decline beyond the established threshold for a minimally important difference were 41% *vs.* 14% (p=0.002) for bowel QoL and 30% *vs.* 17% (p=0.04) for urinary QoL (12). Chapet et al. investigated the injection of HA to preserve the rectal wall during hypofractionated RT for prostate cancer. They first published on the dosimetric gains resulting from the implantation of the HA gel in a cohort of 16 patients (26). Our findings are consistent with the dose and volume reductions following injection of HA, which resulted in significantly limitation of the radiation dose delivered to the rectal wall (26). A subsequent multicenter phase II trial (from 2010 to 2012) included 36 patients with low-risk to intermediate-risk prostate cancer. With regard to acute toxicity, the injection of HA was associated with a mean ± SD pain score (on a 0 to 10 scale) of 4.6 ± 2.3. Grade 2 AEs were reported for 20 patients (19 with urinary obstruction, urinary frequency, or both, and 1 with proctitis) (27).

More recently, in a systematic review and meta-analysis based on 7 studies (1 randomized clinical trial and 6 cohort studies) involving 1011 men (of whom 486 received a PEG hydrogel spacer), the prostate-rectum separation produced by the spacer was sufficient to reduce V70 rectal irradiation (25). The authors of the review also showed that a PEG spacer was associated with fewer rectal toxic effects and better bowel-related quality of life (25).

Lastly, the ProSpace balloon was first investigated by Gez et al. in a multicenter study of 27 patients (20). Although Vanneste et al.’s report in 2017 described filling the ProSpace balloon with iodine contrast solution in 15 cases, Gez et al.’s publication from 2013 did not mention contrast solution. Gez et al.’s results for the dose reduction on rectal volumes were similar to our present results, and acute toxicity was also limited (20). Most of the AEs correspond to mild pain in the perineal area after implantation. Three cases of acute urinary retention resolved in a few hours (20). The results of subsequent studies suggested that although balloon spacers are associated with a signification reduction in rectal doses and are relatively easy to implant, volume loss (i.e. leakage of saline from the balloon) over the course of treatment is a problem (28, 15). Despite the volume loss, the spacing between the prostate and the rectal wall was nevertheless maintained (19). Lastly, in a large, comparative, non-randomized study of patients receiving a gel spacer (n=139) or a balloon spacer (n=264), Schörghofer et al. reported that although use of either spacer reduced the incidence of grade 1 and 3 AEs, grade 3 AEs (rectal perforation) occurred only in patients (n=6) having received the balloon spacer (17). The researchers suggested that this rectal perforation might have been due to the balloon spacer’s rigidity and size (17). In view of the rectal dosimetric gains observed with the balloon spacer and the low frequency of gastrointestinal adverse events during and after implantation, we suggest that this procedure should be used in the next generation of clinical trials on dose escalation as a means of improving the curability of prostate cancer. It would be interesting to investigate the putative benefit of a rectal spacer for hypofractionated dose regimens or intraprostatic dose-boosting procedures with either conventional fractionation or a stereotactic boost, such as the ongoing Simultaneous Integrated Boost for Prostate Cancer study (NCT03664193).

The present study had a number of limitations. Firstly, the inclusion of patients at a single-center (despite an initially multicenter design) means that the results cannot be readily extended to other institutions and settings. Secondly, the study design (i.e. termination once the dosimetric gain had been demonstrated) limited the number of study participants and thus restricted the volume of clinically interesting data on adverse events. Thirdly, we did not include a comparator group, e.g. patients treated with another type of spacer or treated in the absence of a rectal spacer. Fourthly, we lacked some IPSS and QoL data at 24 months post-RT for some patients, and only a small proportion of patients answered the PR25 module’s questions on sexual function (although half the study population received a 6-month course of androgen deprivation therapy). Fifthly, we did not report the rectal spacer balloon’s volume stability (while using daily cone beam CT IGRT quality control insurance during treatment course) during therapy. We didn’t observe any loss of balloon during treatment course.

## Conclusion

A biodegradable rectal spacer balloon was found to be a safe, effective means of obtaining dosimetric gains in the RT of intermediate-risk prostate cancer. The implantation was easy, and the few technical difficulties experienced did not compromise the treatment’s safety or effectiveness.

## Data Availability Statement

The raw data supporting the conclusions of this article will be made available by the authors, without undue reservation.

## Ethics Statement

The studies involving human participants were reviewed and approved by Comité de Protection des Personnes Nord Ouest I, Lille, France; reference: 13/10/2016. Written informed consent for participation was not required for this study in accordance with the national legislation and the institutional requirements.

## Author Contributions

IL, EBr, and DP conceived and designed the study, acquired, analyzed and interpreted, and drafted the manuscript. EBo and MC-D contributed to the statistical analysis and interpretation of data. IL, EBr, DP, EBo, MC-D, EL, and DM helped to supervise the work and revised the article for critical content. All authors contributed to the article and approved the submitted version.

## Funding

The study was funded by Centre Oscar Lambret (Lille, France) and AQUILAB (Loos, France). ProSpace**^®^** biodegradable balloons were provided at cost price by BioProtect Ltd (Tzur Yigal, Israel).

## Conflict of Interest

The authors declare that this study received funding from BioProtect Ltd and AQUILAB. The funder was not involved in the study design, collection, analysis, interpretation of data, the writing of this article or the decision to submit it for publication.

## Publisher’s Note

All claims expressed in this article are solely those of the authors and do not necessarily represent those of their affiliated organizations, or those of the publisher, the editors and the reviewers. Any product that may be evaluated in this article, or claim that may be made by its manufacturer, is not guaranteed or endorsed by the publisher.

## Abbreviations

AE, adverse event; BMI, body mass index; CI, confidence interval; CTV, clinical target volume; DXcc, dose delivered to X cc of the designated anatomic structure; ECOG PS, Eastern Cooperative Oncology Group Performance Status; EORTC QLQ-C30, European Organisation for Research and Treatment of Cancer Core Quality of Life Questionnaire; EORTC QLQ-PR25, European Organisation for Research and Treatment of Cancer Prostate Cancer-Specific Quality of Life Questionnaire; IMRT, intensity-modulated radiation therapy; OAR, organs at risk; OR, odds ratio; PSA, prostate-specific antigen; QoL, quality of life; SD, standard deviation; V70 Gy, volume of the indicated anatomic structure receiving 70 Gy; VX%, volume receiving X% of the prescribed dose.
