# Supplementary material for: Use of a Biodegradable, Contrast-Filled Rectal Spacer Balloon in Intensity-Modulated Radiotherapy for Intermediate-Risk Prostate Cancer Patients: Dosimetric Gains in the BioPro-RCMI-1505 Study
Source: Front Oncol. 2021 Aug 26;11:701998. doi: 10.3389/fonc.2021.701998 (PMC8427159; doi:10.3389/fonc.2021.701998)
Supplement: Supplementary file 1 [file Table_1.docx]

Supplementary Material

# Supplementary Table 1. The BioPro-RCMI-1505 trial’s main inclusion and exclusion criteria.

| **Main inclusion criteria** (applicable upon screening). |
| --- |
| – age 18 or over  – localized adenocarcinoma of the prostate:  ○ intermediate risk, according to D’Amico’s classification  ○ MRI stage <T3  – intensity-modulated radiotherapy  – serum prostate-specific antigen level ≤20 ng/mL before radiotherapy  – prostate volume >15 cc  – no clinical signs of disease progression  – Eastern Cooperative Oncology Group performance status ≤1  – life expectancy ≥10 years  – provision of written, informed consent  – social security coverage |
| Main exclusion criteria |
| – contraindication to the implantation of a ProSpace^®^ biodegradable balloon:  ○ ongoing anticoagulation with a vitamin K antagonist or heparin  ○ immunosuppression or serious chronic diseases (such as heart failure, cirrhosis, and chronic kidney failure)  ○ a history of prostatitis or repeated prostatic resections  ○ past or ongoing colonic or rectal inflammatory disease or lower gastrointestinal infection  ○ an untreated perineal wound  – a history of hormone therapy for 6 months or more. *NB: a short concomitant course (4 to 6 months) of androgen deprivation therapy was not an exclusion criterion*.  – another invasive cancer in the previous 5 years (except treated basal cell carcinoma)  – a history of pelvic radiotherapy  – severe hypertension not controlled by appropriate treatment (systolic arterial pressure ≥160 mmHg and/or diastolic arterial pressure ≥90 mmHg)  – another ongoing cancer treatment  – legal guardianship  – incarceration  – inability to attend or comply with the study’s follow-up visits  – conformal radiotherapy without intensity modulation |

# Supplementary Table 2. Visits and procedures in the BioPro-RCMI-1505 trial.

|  | In the 60 days preceding the first RT session | In the 30 days preceding the first RT session | Within the 21 days preceding the first RT session | | During the RT | After the end of the RT | Follow-up period | Study exit |
| --- | --- | --- | --- | --- | --- | --- | --- | --- |
|  |  |  | Before balloon implantation | After  balloon implantation |  |  |  |  |
| *Clinical examinations* |  |  |  |  |  |  |  |  |
| Medical history, disease history, previous treatments | **🗸** |  |  |  |  |  |  |  |
| Complete clinical examination with assessment of the general condition (ECOG PS) | **🗸** |  |  |  | **🗸** (weekly) | **🗸** | **🗸** | **🗸** |
| IPSS | **🗸** |  |  | **🗸** | **🗸** (midway through treatment) | **🗸** | **🗸** (at 3, 6, 12 and 24 months) | **🗸** |
| *Laboratory tests* |  |  |  |  |  |  |  |  |
| Serum PSA level | **🗸** |  |  |  |  |  | **🗸**(at 3, 9, 15 and 21 months) | **🗸** |
| *Quality of life* |  |  |  |  |  |  |  |  |
| EORTC QLQ-C30 |  | **🗸** |  | **🗸** | **🗸** (midway through treatment) | **🗸** | **🗸** (at 3, 6, 12 and 24 months) | **🗸** |
| EORTC QLQ-PR25 |  | **🗸** |  | **🗸** | **🗸** (midway through treatment) | **🗸** | **🗸** (at 3, 6, 12 and 24 months) | **🗸** |
| *Other examinations* |  |  |  |  |  |  |  |  |
| Tumour evaluation (CT) |  |  | **🗸** |  |  |  |  |  |
| RECIST scoring |  |  | **🗸** |  |  |  |  |  |
| Dosimetric plan (CT) |  |  | **🗸** | **🗸** |  |  |  |  |
| *Treatments* |  |  |  |  |  |  |  |  |
| Treatment of adverse effects related to RT |  |  |  |  | **🗸** (weekly) | **🗸** | **🗸**(at 3, 6, 12 and 24 months) | **🗸** |

RT: radiotherapy; ECOG PS: Eastern Cooperative Oncology Group Performance Status; IPSS: international prostate symptom score; PSA: prostate-specific antigen; EORTC QLQ-C30: European Organisation for Research and Treatment of Cancer Core Quality of Life Questionnaire; EORTC QLQ-PR25: European Organisation for Research and Treatment of Cancer Prostate Cancer-Specific Quality of Life Questionnaire; RECIST: Response Evaluation Criteria in Solid Tumours
